# Efficacy of Local Intralesional Steroid Injection for Pain Relief in De Quervain's Tenosynovitis

**DOI:** 10.7759/cureus.73639

**Published:** 2024-11-13

**Authors:** Usman Hafeez, Muhammad Mannan, Sarmad Khalil, Ali Ullah Ghauri, Faisal Karim

**Affiliations:** 1 Trauma and Orthopaedics, University Hospitals Birmingham NHS Foundation Trust, Birmingham, GBR; 2 Trauma and Orthopaedics, Ghurki Trust Teaching Hospital, Lahore, PAK; 3 Orthopaedic Surgery, University Hospitals Birmingham NHS Foundation Trust, Birmingham, GBR; 4 Orthopaedics and Traumatology, Queen Elizabeth Hospital Birmingham, Birmingham, GBR

**Keywords:** de quervain's disease, efficacy, intralesional steroid injection, pain relief, tenosynovitis

## Abstract

Introduction: Conservative treatment options, such as rest, massage, cold and heat packs, wrist splints, braces, physical therapy, thumb spica casts, and local steroid injections, have been used with mixed results to treat De Quervain's tenosynovitis. Surgical treatment, like releasing the first dorsal wrist compartment, is the last resort for resistant cases of De Quervain's tenosynovitis, exhibiting an efficacy of 91%. However, complications and increased expenses have limited the use of surgical interventions.

Objectives: This study aims to determine the efficacy, measured by the reduction in visual analog scale (VAS) scores over a three-week follow-up period, of local intralesional steroid injections in providing pain relief for patients with De Quervain's disease.

Materials and methods: This study is an analytical case series conducted at the Department of Orthopedics, Ghurki Hospital, Lahore, from March 5, 2020, to September 4, 2020. After receiving approval from the ethical review board of Lahore Medical and Dental College, 91 patients who met the inclusion criteria were enrolled in the study. Informed consent was obtained, and demographic details, including age, gender, affected side, place of residence, occupation (student, office worker, or others), and baseline VAS score, were recorded. For injection preparation, 1 mL (10 mg) of triamcinolone acetonide was mixed with 1 mL (1%) of lidocaine hydrochloride in a 5-cc syringe. The corticosteroid injection was administered using a 24- or 26-gauge needle. Before injection, the area of maximum tenderness was confirmed. The needle was then inserted into the first extensor compartment of the wrist, directed proximally toward the radial styloid process, and aligned parallel to the abductor pollicis longus and extensor pollicis brevis tendons. Swelling of the synovial sheath due to the volume effect was observed. Post-injection, patients were allowed NSAID (nonsteroidal anti-inflammatory drug) tablets for pain relief as needed. The principal researcher administered all injections. Each patient was followed up for three weeks, and efficacy, as defined in the operational criteria, was recorded.

Results: The participants in the study ranged from 25 to 65 years of age, with a mean age of 41.40 ± 10.62 years. The majority of the patients were aged between 25 and 45 years, comprising 60 patients (65.93%). Out of the 91 patients, 56 (61.54%) were male, and 35 (38.46%) were female, resulting in a male-to-female ratio of 1.6:1. In our study, efficacy (in terms of pain relief) of local intralesional steroid injection in De Quervain's disease was seen in 79 (86.81%) patients.

Conclusion: This study demonstrates that local intralesional steroid injections are effective in providing pain relief for De Quervain's tenosynovitis in a Pakistani cohort. This finding aligns with efficacy results from international studies and supports its use as a primary treatment option in diverse populations.

## Introduction

De Quervain's syndrome, often called De Quervain's tenosynovitis, is a condition that impacts the first extensor compartment of the wrist. It affects the tendons of the abductor pollicis longus and extensor pollicis brevis. These tendons help in the thumb movement. Usually, the tendons swell due to attritional wear from actions that require frequent thumb movements, such as texting or typing. That is why it is often called "smartphone thumb" or "Blackberry thumb" [1,2].

People with De Quervain's syndrome often experience pain and swelling on the radial side of the wrist, which can reach the forearm. The pain usually worsens when the thumb is moved, especially when gripping or pinching. Sometimes, a cyst can cause a snapping sensation in the wrist [3].

The clinical history and physical examination of the wrist typically lead to the diagnosis of the condition. A key test is the Finkelstein test, which can show if there is pain and confirm the issue [4]. Treatments often start with simple options such as rest, cold therapy, physical therapy, and corticosteroid injections [5,6]. If these do not work, surgery might be performed to free the tight fascial layer around the tendons, which has an efficacy of 91% [7].

Corticosteroid injections are used often because they help decrease pain and swelling. Studies show that shots of triamcinolone acetonide (a type of steroid) can help relieve pain in up to 83.33% of patients [8]. Another study found a 62% success rate for the same treatment [9,10]. While most of the studies regarding the treatment options for De Quervain's tenosynovitis have been done in Western groups, the data on the effectiveness of these treatments in the local population of Pakistan is missing. This study aims to see the efficacy of corticosteroid injection in relieving pain in people with De Quervain's syndrome in a local group.

## Materials and methods

Study design

This study was an analytical case series conducted at the Department of Orthopedics, Ghurki Hospital, Lahore, from March 5, 2020, to September 4, 2020. A sample size of 91 patients was calculated based on a 95% confidence level, a 10% margin of error, and an expected efficacy of 62.0% for local intralesional steroid injection in De Quervain's disease. The sampling method used was non-probability consecutive sampling, allowing for a systematic analysis of treatment outcomes and contributing factors in the selected patient population.

Sample selection

The inclusion and exclusion criteria are presented in Table 1.

**Table 1:** Inclusion and exclusion criteria of the study

Inclusion Criteria	Exclusion Criteria
All patients with De Quervain’s disease (as per operational definition) of >2 weeks after failed physiotherapy	Patients with chronic medical conditions such as rheumatoid arthritis, gout, diabetes mellitus, and pregnancy
Age 25-65 years of either gender	Previous history of trauma in the first extensor compartment of the wrist
	Previous history of steroid injection in the first extensor compartment of the wrist

Data collection procedure

After approval from the ethical review board of Lahore Medical and Dental College, 91 patients who met the inclusion criteria were included in the study. After obtaining informed consent, demographic features (age, gender, side affected, place of living, occupation (student/office/others), and baseline visual analog scale (VAS) score were noted. For the preparation of injection, 1 mL (10 mg) of triamcinolone acetonide and 1 mL (1%) of lidocaine hydrochloride were taken and combined in a 5-cc syringe for injection. Corticosteroid injection was administered using 24- or 26-gauge needles. The area of maximum tenderness was confirmed before injection. After inserting the needle in the first extensor compartment of the wrist, it was directed proximally towards the styloid process of radius and parallel to the abductor pollicis longus and extensor pollicis brevis tendons. Observation of synovial sheath swelling by volume influence was noted. Post-injection patients were allowed to take nonsteroidal anti-inflammatory drug (NSAID) tablets for pain as required. In all patients, the injection was given by the principal researcher himself. All patients were followed for three weeks, and efficacy (as described in the operational definition) was noted. All data was recorded on a specially designed proforma.

Data analysis procedure

Statistical analysis was performed using IBM SPSS Statistics for Windows, Version 25 (Released 2017; IBM Corp., Armonk, New York). Age, duration of disease, and baseline VAS score were presented as mean and standard deviation. Frequency and percentage were calculated for gender, place of living (rural/urban), side affected (right/left), occupation (student/office/others), and efficacy (yes/no).

Effect modifiers such as age, gender, duration of disease, baseline VAS score, side affected (right/left), occupation (student/office/others), and place of living (rural/urban) were stratified. A post-stratification chi-square test was applied, and a p-value of ≤0.05 was taken as significant.

## Results

The majority of patients were aged between 25 and 45, with 60 individuals (65.93%), and were predominantly male, totaling 56 (61.54%). The majority of 67 patients, accounting for 73.63%, experienced symptoms on the right side. A greater percentage of patients, 61 (67.03%), experienced symptoms for over three months. Baseline VAS scores were primarily between 4 and 6, reported by 51 patients (56.04%). This is presented in Table 2.

**Table 2:** Distribution of patients with De Quervain's disease VAS: visual analog scale

Category	Criterion	No. of Patients	Percentage (%)
Age (in years)	25-45	60	65.93
	46-65	31	34.07
Gender	Male	56	61.54
	Female	35	38.46
Side affected	Right	67	73.63
	Left	24	26.37
Duration (months)	≤3	30	32.97
	>3	61	67.03
Baseline VAS	4-6	51	56.04
	>6	40	43.96

**Figure 1:**
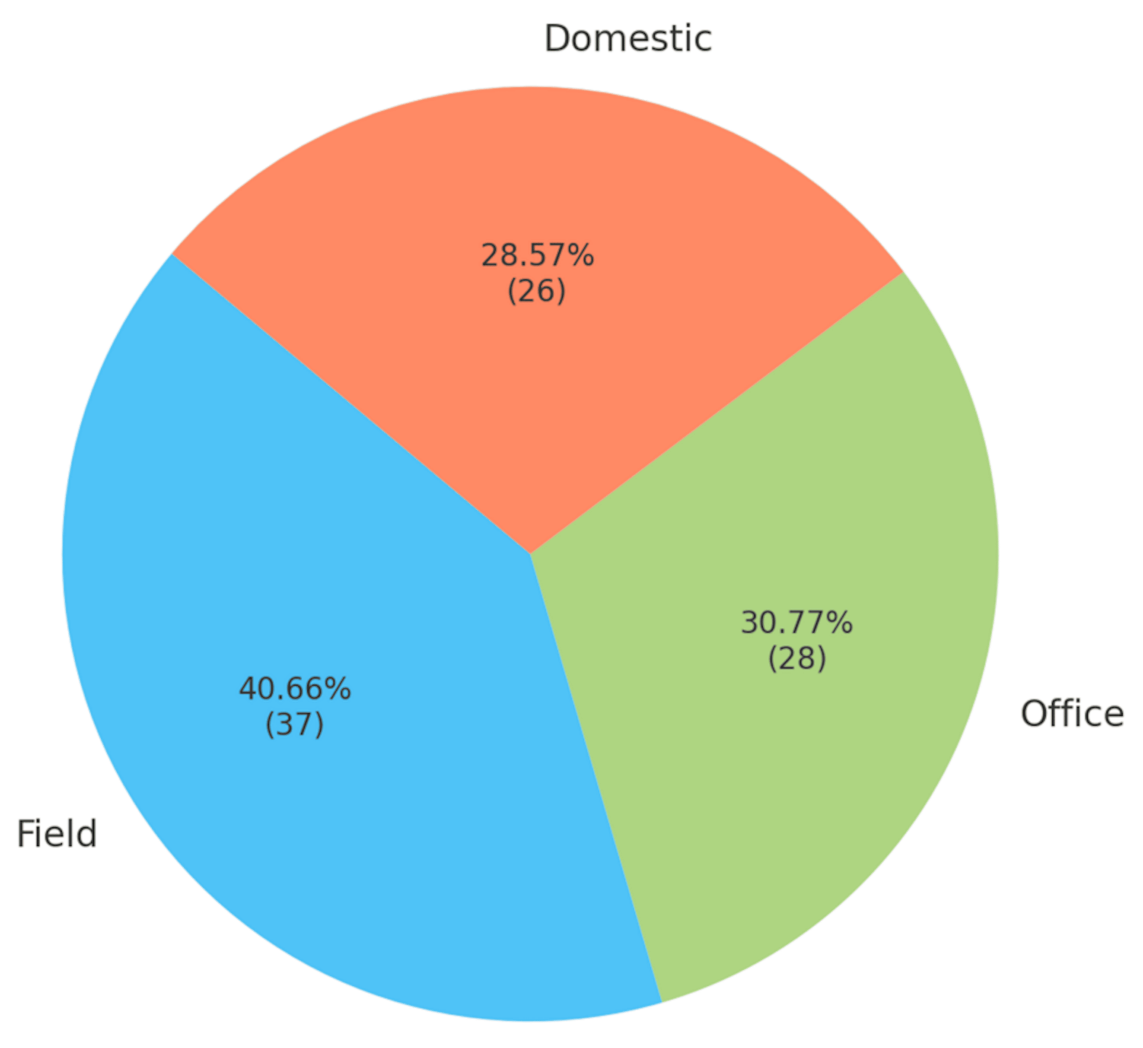
Occupation distribution

Regarding occupation, the largest group was engaged in fieldwork, accounting for 37 patients (40.66%), 30.77% (28) in office roles, and 28.57% (26) in domestic roles (Figure 1).

For age groups, patients aged 25-45 showed a slightly higher efficacy, with 51 patients (85.0%) experiencing efficacy and nine (15.0%) not, compared to the 46-65 group, where 28 (90.3%) had effectiveness and three (9.7%) did not (p = 0.701). In terms of gender, males had 49 (87.5%) reporting efficacy and seven (12.5%) without, while females had 30 (85.7%) with efficacy and five (14.3%) without, yielding a non-significant p-value of 1.000. When evaluating disease duration, patients with ≤3 months duration included 26 (86.7%) with efficacy and four (13.3%) without, whereas those with >3 months duration had 53 (86.9%) experiencing efficacy and eight (13.1%) did not, also with a non-significant p-value of 1.000. Baseline VAS scores of 4-6 revealed that 46 patients (90.2%) had efficacy, while five (9.8%) did not. In contrast, scores >6 showed that 33 (82.5%) had efficacy and seven (17.5%) did not, with a p-value of 0.444, indicating no significant difference. Regarding the side affected, 41 (93.2%) of right-side patients reported efficacy compared to three (6.8%) without, while for the left side, 38 (80.9%) experienced efficacy versus nine (19.1%) without, yielding a p-value of 0.153. In terms of occupation, 31 (83.8%) field workers reported efficacy, while six (16.2%) did not; office workers reported 25 (89.3%) and three (10.7%) did not; and domestic workers reported 23 (88.5%) and three (11.5%) did not, resulting in a p-value of 0.776. Overall, 79 (86.8%) of the patients experienced efficacy, while 12 (13.2%) did not, indicating a broad efficacy across these categories without statistically significant differences.

The efficacy of this study is defined as a reduction in VAS scores, specifically a decrease of at least 50% from baseline measurements, which indicates meaningful pain relief and improvement in symptoms (Table 3).

**Table 3:** Stratification of efficacy VAS: visual analog scale

Category	Criterion	Efficacy (Yes)	Efficacy (No)	p-value
Age group	25-45	51	9	0.701
	46-65	28	3	
Gender	Male	49	7	1.000
	Female	30	5	
Duration of disease (months)	≤3 months	26	4	1.000
	>3 months	53	8	
Baseline VAS	4-6	46	5	0.444
	>6	33	7	
Side affected	Right	41	3	0.153
	Left	38	9	
Occupation	Field	31	6	0.776
	Office	25	3	
	Domestic	23	3	
Overall efficacy	-	79	12	-

## Discussion

De Quervain's syndrome can be treated with both surgery and conservative methods. The surgery to release the first extensor wrist compartment works well, with success rates up to 91%. However, the surgical options are associated with complications such as scars, hypersensitivity, and nerve issues. Because of these complications, non-surgical treatments are preferred, especially corticosteroid injections, as they are less invasive and effective pain relief [11,12].

A meta-analysis of six randomized controlled trials (RCTs) showed that corticosteroid injection with thumb spica splints worked better than either treatment option alone [13]. In another study by Mardani-Kivi [11], corticosteroid injection alone had an efficacy of 76%, but adding a thumb spica cast raised the efficacy to 93%. These studies highlight the importance of using more than one method in the treatment of De Quervain's tenosynovitis [14].

Additionally, using ultrasound to localize corticosteroid injection has been shown to improve the localization of the inflamed tendon and injecting targeted injection, and this has increased the efficacy to 91% [15]. In our study, we found that local corticosteroid injection had an effectiveness of 86.8% (79), which validates earlier findings of efficacy between 83% and 90% [16]. Triamcinolone's slow absorption and long duration of action in the tendon sheath likely help its effectiveness, especially in refractory cases of De Quervain's syndrome [17].

While corticosteroid injections are generally safe, potential side effects include short-term pain increase, skin depigmentation, and local muscle wasting. It is important to discuss these possible complications with patients before starting treatment [18,19]. Notably, none of our patients experienced any of these side effects.

Other methods, like acupuncture, have also been investigated. One trial showed significant relief in pain and improvement in function for patients who received either acupuncture or corticosteroid injections. However, corticosteroid injections yielded slightly better overall outcomes [4].

Limitations

The study has several limitations. The small sample size makes it hard to apply the findings to a general population. Having a large sample size would give insight into the efficacy of corticosteroid injection in the local population. Also, there was no long-term follow-up for the patient, which did not allow us to see the long-term efficacy of the treatment. Doing the study in just one place limits its wider applicability to other centers as the method of preparation and injecting the corticosteroid might vary between different centers, and patient backgrounds can vary in different areas. Thirdly, there was no control group to compare the efficacy of the treatment.

## Conclusions

This study provides evidence supporting the efficacy of local intralesional steroid injections in relieving pain associated with De Quervain's tenosynovitis among patients in Pakistan, aligning with findings from similar studies conducted in other populations. While previous studies have shown the effectiveness of corticosteroid injections for this condition, this study contributes localized data, confirming similar benefits within a Pakistani population. This supports the broader applicability of corticosteroid injections as a first-line treatment for De Quervain's tenosynovitis in diverse Pakistani populations. Further research involving larger, multicenter samples across varied regional populations would enhance the generalizability of these findings and inform treatment standards. This conclusion is based on quantitative data collected using VAS scores. Efficacy is defined as a reduction in VAS scores of at least 50% from baseline, indicating significant pain relief in post-injected patients.
